# Sensors Systems for the Automation of Operations in the Ship Repair Industry

**DOI:** 10.3390/s130912345

**Published:** 2013-09-13

**Authors:** Pedro Javier Navarro, Juan Suardíaz Muro, Pedro María Alcover, Carlos Fernández-Isla

**Affiliations:** 1 Universidad Politécnica de Cartagena, TIC, Plaza del Hospital, 1, Cartagena 30202, Spain; E-Mails: pedroj.navarro@upct.es (P.J.N.); carlos.fernandez@upct.es (C.F.-I.); 2 Universidad Politécnica de Cartagena, DTE, C/ Doctor Fleming,s/n, Cartagena 30202, Spain; E-Mail: juan.suardiaz@upct.es

**Keywords:** sensor systems for ship repair industry, grit blasting automation, vision sensors, image processing surface fault detection

## Abstract

Hull cleaning before repainting is a key operation in the maintenance of ships. For years, a method to improve such operation has been sought by means of the robotization of techniques such as grit blasting and ultra high pressure water jetting. Despite this, it continues to be standard practice in shipyards that this process is carried out manually because the developed robotized systems are too expensive to be widely accepted by shipyards. We have chosen to apply a more conservative and realistic approach to this problem, which has resulted in the development of several solutions that have been designed with different automation and operation range degrees. These solutions are fitted with most of the elements already available in many shipyards, so the installation of additional machinery in the workplace would not be necessary. This paper describes the evolutionary development of sensor systems for the automation of the preparation process of ship hull surfaces before the painting process is performed. Such evolution has given rise to the development of new technologies for coating removal.

## 1. Introduction

**Figure 1.**
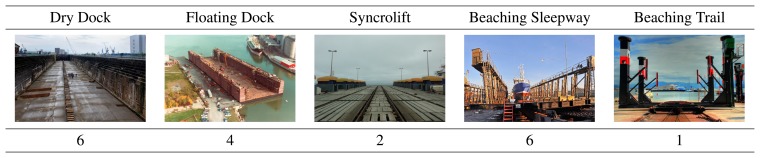
Typology of the Spanish shiprepair yards: Number of Shipyards working in every Beaching Method.

The ship repair industry in Spain represents a turnover of around EUR 500 million a year and employs 22,000 people. The largest turnover generated in the Ship Repair Industry comes from the periodical maintenance operations that are usually performed on ships to guarantee their safe and efficient sailing conditions. Despite the great importance of the maintenance operations, most of the cleaning works are carried out directly by operators, almost in a manual way. The maintenance works are usually programmed so they coincide with the ship hull cleaning and repainting operations, which are performed every 4 or 5 years and provide a substantial work load to the Spanish ship-repair yards (around 2 million square meters are repaired and repainted in Spain every year). Figure 1 shows the different Beaching Methods and the number of shipyards that make use of these methods in Spain.

**Figure 2.**
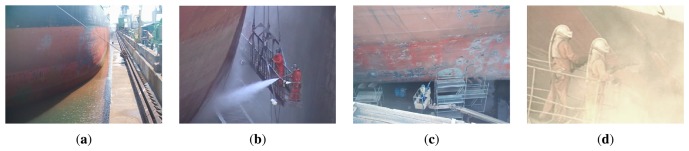
The maintenance works of a ship hull. (**a**) Beaching; (**b**) Ship Hull Desalination; (**c**) and (**d**) Grit-blasting cleaning process.

The maintenance works of a ship hull start with the beaching operation (Figure 2a). Then, the ship hull desalination process starts (Figure 2b), followed by the cleaning of damaged areas by blasting the hull with high pressure grit. (Figure 2c,d). This process is called grit blasting within the ship repair industry. The grit blasting process entails two cleaning methods: full blasting and spotting. The full blasting cleaning method is used to clean the ship hull as a whole. The spotting cleaning method is used for cleaning specific damaged areas (sometimes these areas have the size of a coin). Given the current cuts produced in the ship owners budget, the spotting cleaning method is the most demanding method for the ship hulls maintenance operations, not only because it requires less working time than the full blasting method, but because it needs a lower amount of grit (which leads to lower costs and smaller environmental impact).

The most widely used and most preferred technique for cleaning ships hulls [1,2] consists of open-air blasting of the hull with metallic grits. This technique achieves the optimal standardized SA $2\frac{1}{2}$ [3] surface finish for the hull, which assures good paint adherence and prolongs the periods between further repainting. The ultra high pressure (UHP) water jetting [4] does not achieve the same surface finish. Furthermore, the robotized systems based on this technology are too expensive to be widely accepted by shipyards.

These cleaning and repairing works performed on the surface of the ship's hull have certain issues though:

(1) Uneven hull surface, especially those areas of the hull known as “fine” areas (areas with deep edges in the ship's forward, keel and propellers location), and “bottom” areas (lower parts of the ship hull presenting very warped areas). All these defects make it difficult to access to some of the surfaces of the ship hull that must be repaired.
(2) The grit blasting operation is carried out in open air. Therefore, while such operation is being performed a big cloud of dust is produced containing traces of abrasive substances, heavy metals and biocidal products. The lack of confinement also produces a high noise pollution with sustained level of noise that reaches 120 dB.
(3) The powerful reaction produced by the high pressure generated by the grit blasting tools used during the cleaning process.
(4) Presence of many obstacles within the work area limits (scaffolds, cranes, rails, wires, hoses, *etc.*).

All these issues turn the workplace into a very difficult place to work. Operators must protect themselves from different types of pollution by wearing very heavy and uncomfortable protective suits, respirators and earplugs while they perform tasks that demand great physical efforts and unusual and uncomfortable positions to overcome the powerful reaction generated by the grit guns.

It is necessary to create better work conditions that help to reduce the risks taken by the operators and the harmful environmental impact produced at the workplace. A good way to start would be to automate all the cleaning works. However, such automation is quite difficult to apply on account of the abundant obstacles in the workplace and the great variety of surfaces that require repair works. Besides, this automated system must be sturdy enough to avoid constant maintenance works regardless of the described challenges of the workplace.

For some time, robots for cleaning large vertical surfaces either with water [5] or with grit [6] have been available, resulting in a very high standard of work although at a substantial cost. In addition to this, robotic solutions based on robotic climbers have existed for some time. However, they all use high-pressure water jetting technology, which curbs their use for the reasons previously mentioned. Among the systems currently available, the system developed by Ultrastrip Systems, Inc. is worth mentioning [7]. This vehicle is built of aluminum and titanium and is attached to the hull by the combined use of a magnetic head and a vacuum system. Perhaps it is the most efficient system but it is very expensive and uses water jetting. The Hydro-Crawler system developed by Dans Vandteknik [8], the HydroCat system of Flow International Corporation [9], and Octopus system of Cybernetix [10] are also worth mentioning. These systems entail three major disadvantages: (1) the lack of competitiveness because of the high costs involved; (2) their dependence on high pressure water (these systems do not accept grit blasting); and (3) their exclusion of the spotting method among their cleaning techniques.

Therefore, we have chosen to apply a more conservative and realistic approach to this problem. There are several solutions that have been designed with different automation and operation range degrees. These solutions are fitted with most of the elements already available in many Spanish shipyards, so the installation of additional machinery in the workplace would not be necessary. Different prototypes have been designed according to the different features of the surfaces to be cleaned. The addition of systems based on artificial vision equipment to carry out the corresponding inspection and cleaning tasks is a remarkable improvement for the operators who from now on will be able to:

(1) Visualize on a display by means of a camera the image of the area to be cleaned.
(2) Select the cleaning spots directly on the ship hull display.
(3) Control the consumption of grit, energy, cleaned surface, ship model, *etc.*

As explained throughout this paper, there is a ships inspection sensor system that has been developed for automating the cleaning process of the ship's hull. The main contribution of this sensor system is the addition of built-in artificial vision equipment. Nowadays there is no shipyard that is fitted with such technology to clean the surfaces of the ships.

Before applying the definitive system, several prototypes had to be developed in the first place. The first prototype, described in the following section, is made up of a platform lift fitted with a cleaning head. The sensors of this prototype must be improved and the platform lift needs to be adjusted in order to prevent the oscillation produced by its changes of position from affecting the structure. The second prototype, described in Section 3, has been fitted with a elevation tower of a great height, available at the shipyard, and with a platform with sensors that supports the cleaning head and places it on the corresponding position with accuracy and speed: this device has an artificial vision system and a wide sensor network that provides the system with information of its relative position with regard to the ship. The different vision algorithms used in this built-in system are described in this section. The third prototype, explained in Section 4, has definitively improved the design of the cleaning systems. This new prototype has the performance of the second prototype and allows working on larger surfaces of the ship and performing complete full blasting operations at a very competitive speed, if compared with the speed achieved by the manual work of the operators. This paper ends with a section of conclusions.

## 2. A First Approach to an Automatic Cleaning System of Ship Hulls

The design of the different inspection and cleaning systems that have been developed and that are now described in this paper have been carried out in accordance with the following specifications:

(1) The robots must be cost-effective.
(2) The systems must be designed to work with high pressure water and grit and to produce a finishing in the ship surfaces that complies with the required Quality Standards (standard SA).
(3) The designed systems must be fitted with an automatic residue collection device. This way, the impact that the cleaning processes have on the environment is drastically reduced.
(4) The new developed systems must allow the cleaning of the whole surface of the hull (full-blasting) and the cleaning of specific damaged areas of the ship hull (spot-blasting).

On this first approach, the system is composed by two main subsystems:

(1) A mechanical subsystem where the cleaning head, sensors and actuators were mounted.
(2) An automated control subsystem for manoeuvring the robot along the hull surfaces.

The mechanical subsystem is composed of the following functional modules:

- **Elevation platform.** This system consists of a hydraulic elevation platform, whose minimum height is 800 mm and range of elevation is 2,500 mm. Therefore, it is able to clean the fringe of the ship between 800 mm and 3,300 mm high. For the rest of the surface to be cleaned, an additional height of 2,500 mm is added using a complementary structure in such a way that one could sweep the fringe of the hull between 3,300 mm and 5,800 mm.
- **The control unit.** The Control Unit is a sensor subsystem that is able to place the elevation platform and the cleaning head in any desired position. The control unit monitors the movement of all the engines of the elevation platform and the cleaning head arm.

**Figure 3.**
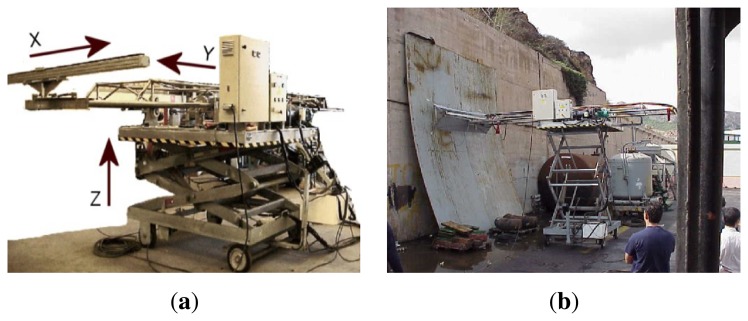
First approach to automatic of cleaning of ship hulls.

This system allows the operator to monitor the Control Unit by means of a Control Panel. The operator enters the location where the cleaning head must be positioned and then controls the opening and closure of the grit flow that must be blasted on the surface to be cleaned. This prototype is fitted with a closed circuit television (CCTV) equipment that helps the operator to command the device remotely, avoiding the hostile cleaning area. These commands are generated using a very primitive electromechanical interface based on buttons, switches, indicators and displays. This system, shown in Figure 3a, controls the movement of the different elements that constitute the robot and cleaning head. Therefore, it elevates or descends the platform, moves forward or backwards the arm and locates the cleaning head in the desired position.

The sensorial capacity of this first developed system is limited and is basically composed of limit switches, encoders and actuators of the elevation hydraulic system and the drive system. The control system is based on a PLC supported with software that produces repetitive movements on the surface to be cleaned (see Figure 3b).

## 3. Sensorised Robotic Systems for Cleaning Ship's Hulls

The search of solutions to perform full or spot blasting on different work surfaces (“fine” and “bottom” areas, and markedly flat and vertical areas) has led to the development of different sensor and robotic systems [11]. These systems have a similar architecture consisting of:

(1) one primary positioning structure;
(2) one optional secondary structure mounted on the primary structure;
(3) one cleaning system;
(4) one smart control and inspection system to control the different elements within the cleaning system.

**Figure 4.**
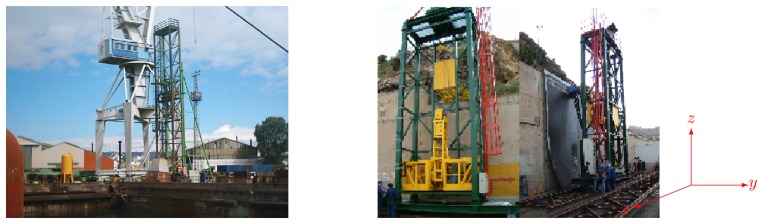
Primary Structure Vertical Towers.

**Figure 5.**
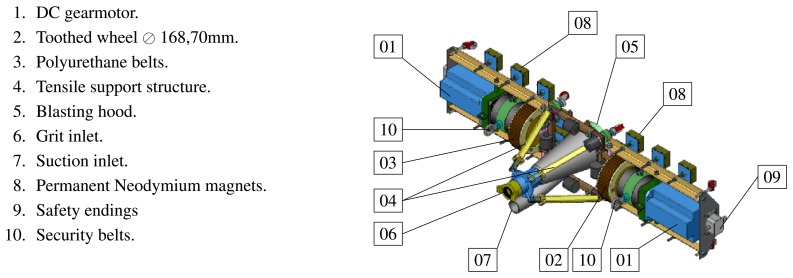
Robotic climber.

**Figure 6.**
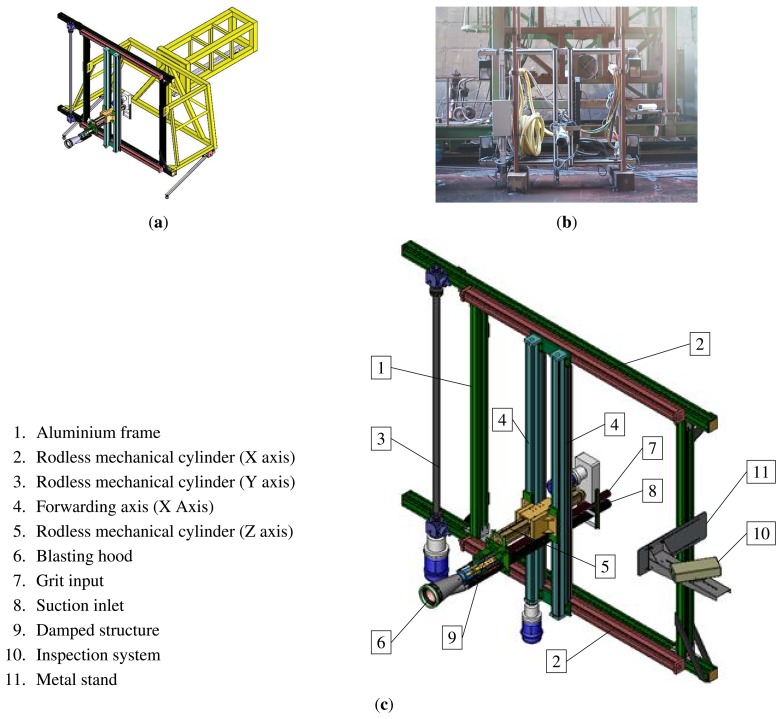
XYZ Table design. Secondary structure. (**a**) 3D view of the XYZ Table; (**b**) Developed XYZ Table; (**c**) Elements of the XYZ Table.

**Figure 7.**
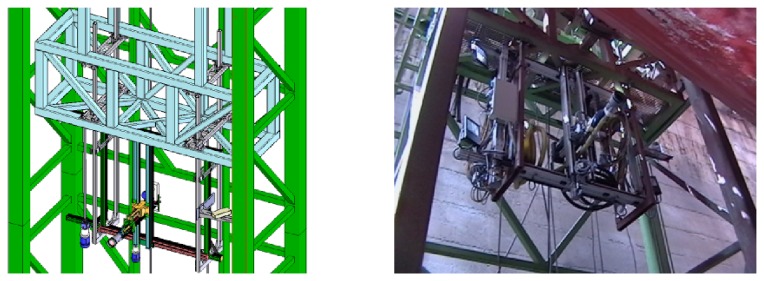
Attachment of the secondary structure.

**Figure 8.**
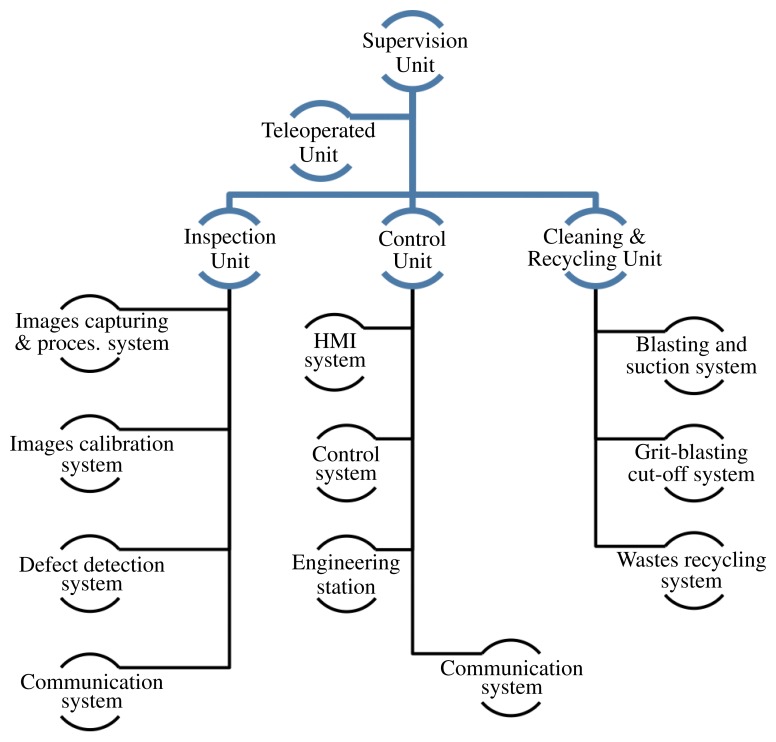
Elements of the visual inspection and control system.

At this stage of development, two primary structures have been used: robotic vertical towers and robotic climbers; and one single secondary structure, called XYZ Table. Also, two potential cleaning heads have been used: one turbine and one grit blasting mouthpiece with a confinement hood. A brief description of these elements is given below:

- **Vertical Towers:** the elevation tower described before was affected by strong oscillations every time the cleaning head changed its position. Besides, this tower required special infrastructures to be moved within the shipyard facilities. In order to give a solution to these problems, two vertical towers fitted with mobility infrastructures and commonly used in shipyards were used. Both towers have three degrees of freedom; they are moved on rails and they have a load capacity of 500 kg. The first tower (Figure 4a) is 30 m high (*Z*-axis); it is placed on a dry dock and it can move on rails along 300 m (*X*-axis). This tower allows a variation in the assembly of up to 2 m in *Y* -axis during the assembly process.
  The second tower (Figure 4b) relies on a Synchrolift system; it is 12 m high (*Z*-axis) and it can move on rails along 100 m in *X*-axis. As it happens with the other tower, this tower allows a variation in the assembly of up to 2 meters in *Y* -axis. This second tower also has two degrees of freedom to position the device according to the shape of the hull.
- **Robotic climber:** The robot climber consists of a vehicle (see Figure 5) that adheres magnetically to the hull and that is capable of moving at a speed of 0.5 m/s without gritting and 0.2 m/s when blasting grit [12]. This vehicle has a load capacity of 10 kg and it is used mainly to gain access to areas of the surface of the ship where obstacles and uneven areas of the hull make it difficult to access the remaining primary or secondary positioning structures.
- **XYZ Table** (Figure 6) This is the secondary structure that has been used to perform the spotting operations. The XYZ Table has a useful surface of 2 × 1.6 m^2^ and it has three degrees of freedom according to *X*, *Y* and *Z* axes. This system is composed of a metallic structure that is able to position any cleaning head on a useful surface with a ± 2 mm accuracy. This device moves at a speed of 1 m/s to take the required positions to carry out the grit blasting process, and at a speed of 0.2 m/s to perform the actual grit blasting process.
  Figure 4 shows the vertical towers. The secondary positioning structure (XYZ Table) is attached to the vertical towers. Figure 7a shows a diagram of the attachment procedure; Figure 7b shows a picture of the attachment. The positioning of the XYZ Table on the surface to be cleaned is carried out by the shipyard operators, who are experts on the use of the primary structure.
- **Cleaning heads:** Two types of cleaning heads were used for our prototype; one head was fitted with a turbine to work on wide and vertical surfaces and to apply the full blasting cleaning method; the other head, which we called Blasting and Suction Hood, is smaller and is made up of two concentric cylinders (see Figure 6). The inside cylinder is used as a nozzle to blast grit on the hull and to perform the corresponding cleaning tasks on its surface. The outside cylinder is used to suck the impurities produced during the grit blasting process. A series of bristles placed on the external edge of this cylinder catches the impurities and improves the support of the cleaning head on the surface of the hull.
- **Visual inspection and control system:** The automation of a system designed to select different areas within a specific surface demands the development of a visual inspection device. In order to get a correct position on the selected areas of the surface, to adjust the grit flow to get the desired finishing on the surface, to update the database linked to the ship where the cleaning tasks are being performed and to complete the whole cleaning process, the device needs to be connected to a control system that synchronizes the different elements in order to activate every single cleaning task in the right moment. The final system obtained is subdivided into different autonomous units to provide it with greater robustness. These autonomous units are the following: (1) supervision unit; (2) inspection unit; (3) control unit; (4) remote operation unit; (5) wastes cleaning and recycling unit. Figure 8 shows the elements every unit is composed of. This figure also shows the relations and communication links between the different units.

The following Sections (3.1 to 3.5) describe the units that form the visual inspection and control system. Finally, Section 3.6 presents a brief evaluation about the performance of the XYZ Table equipped with the visual inspection system.

### 3.1. Supervision Unit

The Supervision Unit is in charge of starting the process and interacting with the operator that enters the cleaning tasks commands. This unit manages the communication with the rest of the units of the visual inspection and control system so it is able to follow the commands entered by the operator. The graphic interface of this unit is programmed in C++ and it allows the selection of any of the operation modes of the system:

- **Supervisor mode.** This operation mode helps the user to redefine the setup parameters of the different units involved in the cleaning process.
- **Operating mode.** This operation mode activates the cleaning process with the parameters previously defined in the Supervisor mode. These setup parameters cannot be modified in the other units. This is the default operation mode under normal work conditions.

### 3.2. Inspection Unit

The graphic interface of the Inspection unit provides the operator with visual information of the area to be cleaned after the XYZ Table has been correctly positioned by the primary structure. This unit presents two operation modes:

- **Manual or Supervised mode.** When this mode is activated, the Inspection unit captures an image of the surface to be cleaned and select the areas where the spotting method is going to be applied. Before selecting these areas, the vision system calibrates and rectifies the captured image and displays a view similar to the view that the operator would get if he were in the XYZ Table. After selecting the spotting areas, the grit blasting process starts automatically, thanks to the interaction between the sensors and actuators the robotic system is made up of. To select the spotting areas, the operator only has to click the mouse.
- **Automatic mode.** When this mode is activated, the inspection system automatically searches for defects or damages on the surface of the hull, by means of an image processing algorithm specifically designed for this purpose, and automatically selects the areas where grit blasting is required. The operator could deselect any area marked by the system, and select those areas actually rejected by the system. The default algorithm used for detecting defects is the thresholding based on the Unsupervised Background Estimation (UBE) algorithm. After the confirmation of the cleaning areas by the operator, the system proceeds as if the Manual mode was activated.

The inspection unit is made up of four systems that contain vision algorithms and communication protocols. These systems are the following: (1) image capture and processing systems; (2) image rectification systems; (3) defect detection system based on the UBE algorithm; and (4) communication system.

#### 3.2.1. Image Capturing and Processing System

In order to facilitate the capture of images, the four vertices of the XYZ Table have been fitted with four halogen lamps. The total power of this illumination system is 1,000 W, it must be very efficient during the night shifts and it should provide enough light to those areas of the ship that are poorly illuminated. The four halogen lamps can be adjusted from the Supervision unit in order to compensate the shades and light reflections produced by the metallic surfaces. Such shades and lights severely affect the performance of the image processing algorithms used for the automatic detection of defects.

**Figure 9.**
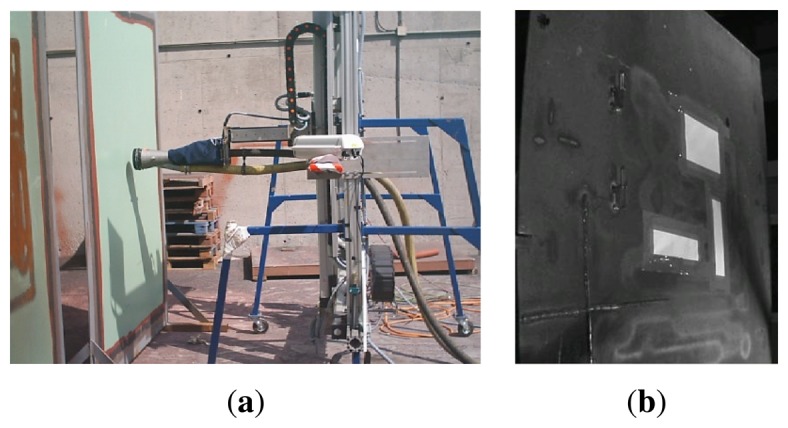
XYZ Table. (**a**) Position of the camera in the left frame of the table; (**b**) An example of a distorted image.

The images are captured by a digital color camera with IEEE1394 interface and high resolution (1,600 × 960 pixels). The camera has been placed on the left side of the XYZ Table frame (Figure 9) to prevent the bouncing grit from damaging it and the elements of the XYZ Table arranged on the front edge stand on the field of vision of the camera. These restrictions, together with the size of the area to be inspected and the distance of the cleaning device with regard to the ship hull (between 1 and 2 m), demand the use of a wide-angle lens that satisfies the requirements of the image capture system. The optical aberrations together with the deformations produced by the position of the camera (as shown in Figure 9b) forced the calibration of the images before processing them. At first, the calibration process was applied in dot patterns images captured at different distances between the hull surface and the camera placed on the XYZ Table. However, this process needs a group of images for every potential new profile of the surface to be inspected, which is not possible in practice. For all these reasons, an Images Correction Affine Warping system has been developed, as described in detail in Section 3.2.2.

The image processing system consists of an Industrial PC, one IEEE1394 frame-grabber, connected to the capturing system of the camera, and a PROFIBUS-DP communication card through which the image processing system communicates with the control unit. A Matrox Pulsar board with an IEEE1394 interface as the image acquisition system frame-grabber has been selected, which is connected to the digital color camera. The Matrox Pulsar board supports image captures up to 30 frames per second and it is capable of sustaining transfer rates of up to 60 MB/s between the acquisition section and the host PCI system (depending on the host computer used). Thanks to the use of a specific library (MIL, Matrox Imaging Library), it also allows fast image processing using embedded processing in the board. The Matrox Pulsar is installed in the Industrial PC and it is controlled using a specific graphical interface designed for that purpose.

In the industrial PC is also installed a PROFIBUS-DP communication card. This communication card allows to synchronize the image acquisition times previous to the inspection process and, once the cleaning areas has been selected, this communication card allows that the image processing system send all the information needed for controlling the movements of the cleaning elements to the control unit.

The management of the frame-grabber for capturing images is carried out by MIL imaging processing libraries with support for C++. Among others, the image processing system performs the following functions: (1) to display a constant video of the cleaning process and the ship conditions; (2) to capture images of the area to be inspected; (3) to share the graphic interface with the operator; (4) to send the spotting areas image to the control unit; (5) to control the system alarms; and (6) to act as an engineering station in the control unit programming.

#### 3.2.2. Image Calibration Affine Warping System

To solve the image correction problem previously described (Section 3.2.1.), we developed an image correction technique, which dynamically creates a pattern independent from the surface geometry and from the distance between the camera plane and the ship hull plane. For that, we slightly modified the prototype, by adding a high brightness LED diode to the blasting nozzle, visible to the camera from any position. We modified the procedure as well, by including a previous phase of creating a rectangular grid of control points located on the hull surface to be treated, and registered in the image plane. The projections of the control points onto the image plane determine a quadrilateral mesh, which is then transformed by a suitable warping algorithm. This algorithm constructs an image, based on the side image captured by the camera, consisting of a mosaic of rectangles provided by the individual transformation of each quadrilateral in the source image.

Our aim is to create a working image where every single point corresponds to a point in the ship surface, and where after selecting any point in the image, the blasting nozzle is able to place itself opposite the corresponding point in the ship surface. For this purpose we have developed a piecewise affine warping transformation. In order to create this working image we use the high brightness LED diode; the robotized system lights on every time the nozzle touches the ship surface in each of the movements described in the previous point. Every time the LED lights on, the camera takes a picture and processes the image to find the new point of the grid where the LED diode light is located. This sequential process is performed as many times as points we need in the resolution of our grid. Each grid point is acquired using an image processing algorithm over each picture where the LED was lit on. Every LED position has its corresponding point in the grid. The distance between two consecutive points is a fixed value, both on the hull surface as in the final corrected image, and these distances are known. It is possible to locate, on the hull surface, any point selected over the final image by means of the warping algorithm previously indicated.

**Figure 10.**
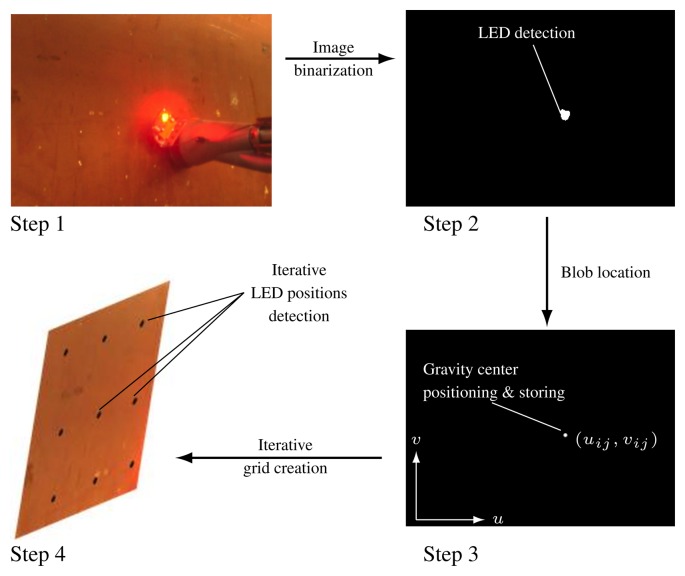
Grid creation using a high brightness LED. The automated system controls the cleaning head, with a high brightness LED placed on it, to position it on each point of the pre-established grid. When the desired (*X*, *Y*) position on the grid is achieved, the automated system moves the cleaning head on the *Z* axis until the hull surface is reached. Then, the LED is switched on, and the vision system acquires an image (step 1). A binarization using a dynamic threshold is performed over the acquired image (step 2). The output of the image binarization is the set of pixels where the LED position is located on the hull surface. A next step (step 3) is performed to find the gravity center of the set of points of the LED position. This point will be labeled as the grid point on the hull surface associated to the equivalent point on the XYZ Table. After performing these steps iteratively over all the (*X*, *Y*) grid points of the XYZ Table (step 4), the full equivalent grid points on the camera plane are obtained. These transformed points will be the key points for the warping transformation.

**Figure 11.**
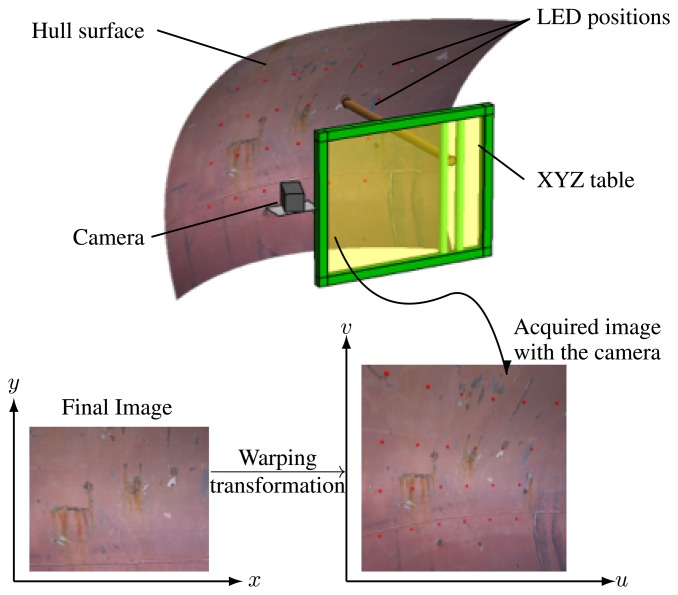
Diagram with all the elements that take part in Warping transformation.

Thanks to the chosen wavelength of the high power LED, the image segmentation is rather simple. Different classic segmentation algorithms have been tested [13–16]. This value is subsequently used to calculate the local threshold of each area of the image, making it possible to determine whether or not a pixel belongs to the LED point projected onto the hull. The method has been tested against other classic thresholding methods and has proved highly stable in variable lighting conditions. Figure 10 shows the thresholding process over a real image. To find the final position of those points on the ship image taken by the camera, we just need to superimpose the points' image and the ship image. In Figure 11 we can see a diagram with all the elements that take part in the transformation.

#### 3.2.3. Defect Detection System Based on the UBE Algorithm

The defect detection system addresses two major problems. The first problem derives from the capture of images since it is typically carried out in open air and under highly variable atmospheric and lighting conditions. This is an aspect that will very much influence the method that is designed for defect detection as described in the following section. The second one concerns computational cost, derived from the spotting process requirements. The image processing system should provide the 3D coordinates of the points to be cleaned in a reasonable time period.

With these requirements, a new thresholding method has been proposed for the detection of defects, which has been denominated UBE (thresholding based on Unsupervised Background Estimation). Method has been divided into two stages. In the first stage a global calculation is carried out on the images to estimate a parameter that has been called a Histogram Range for Background Determination (*HRBD*). This will serve as a reference during the local calculation. In the second stage, using this parameter as a starting point, the image is binarized following the steps detailed below.

##### First Stage: Determination of HRBD and Sensitivity

This method allows automatically estimating the foreground/background ratio by analyzing the histogram of the image.

After a thorough analysis on a group of 240 images acquired under different lighting conditions, it was concluded that the greater part of the background was judged to be situated between points *V_i_* and *V_d_*. The difference between these two values has been called the *HRBD*-Histogram Range for Background Determination.

Once the *HRBD* has been calculated, a sensitivity value (*S*) is calculated so that the calculation of the local threshold in the second stage of the method can be fine-tuned; this value is a ratio determined by the number of histogram entries different from zero (*N_xs_*) divided by the *HRBD*. This value computes the ratio between the total size of the histogram with values different from zero and the estimated size of the background, *HRBD*, and is calculated according to the following Equation:

$$S=\frac{N_{xs}}{\text{HRBD}}$$

$$r=\text{maxLGL}-\text{minLGL}\{\begin{array}{l} r\geq \text{HRDB},\;t=\text{maxLGL}-\text{HRDB}/S\\ r<\text{HRDB},\;t=(\text{maxLGL}+\text{minLGL})/2 \end{array}$$

being *r* the range of the neighborhood of the pixel, which is determined as the difference between the maximum and the minimum of the Local Grey Level (*LGL*).

**Figure 12.**
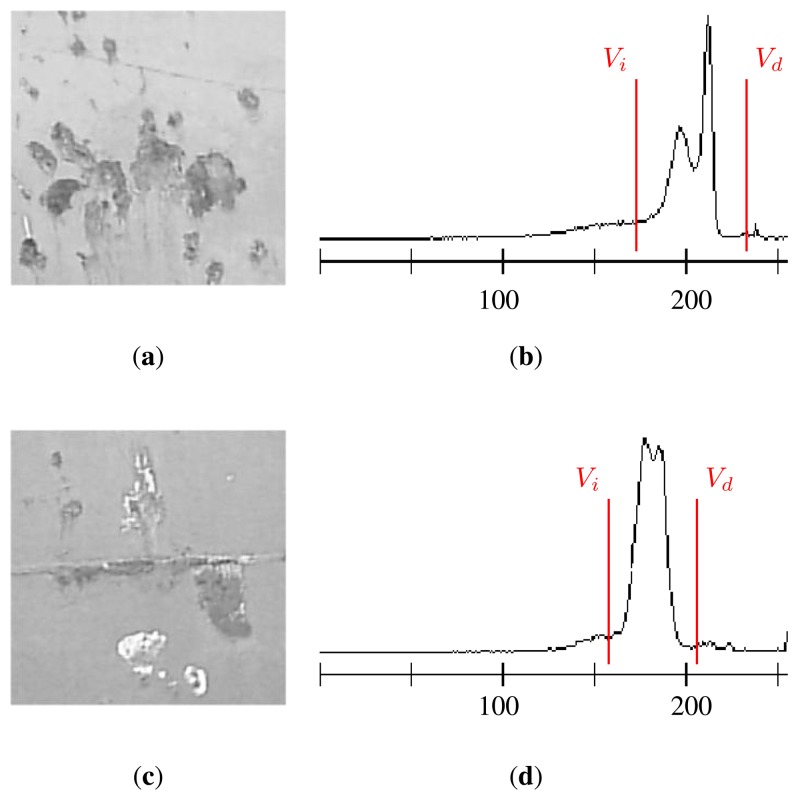
Images with defects and different illumination conditions (**a**), (**c**), and their corresponding histograms and determination of the *HRBD* (**b**), (**d**). (**a**) Image H1; (**b**) *HRDB* = 58, *S* = 179/58 = 3.08; (**c**) Image H6; (**d**) *HRDB* = 48, *S* = 168/48 = 3.04.

Figure 12 shows graphically the results of calculating the *HRBD* = *V_d_* − *V_i_* on the histograms of two images from the case study (H1 and H6). In the figures, note how the points that allow the calculation of the *HRBD* are located on both sides of the most significant distribution. An intensive search for the significant minima both to the left (*V_i_*) and to the right (*V_d_*) of the main distribution was performed to determine the *HRBD* automatically, in which a significant minimum was taken to be the nearest minimum to the left or right, respectively, of the maximum of the histogram. Also shown are the numeric values derived from calculation of the *HRBD* and the sensitivity.

##### Second Stage: Segmentation of the Image

Once the *HRBD* has been calculated, the image is scanned pixel by pixel to determine which pixel belongs to the background and which does not. To do this, the method analyzes the neighborhood of each pixel; this neighborhood is formed by a window of size *k* × *k* centered on the pixel in question, *k* being a natural odd number greater than one and smaller than the dimensions of the image. The neighborhood analysis determines the value of the local threshold (*t*) for the *k* pixel binarization.

To determine the local threshold of each pixel *t*, the following equations are used:

If the current value of the pixel is equal to or greater than this threshold, it is considered to be a background pixel (grey level 255); otherwise it is considered as a pixel associated with the defect and is allotted a grey level value of zero.

**Figure 13.**
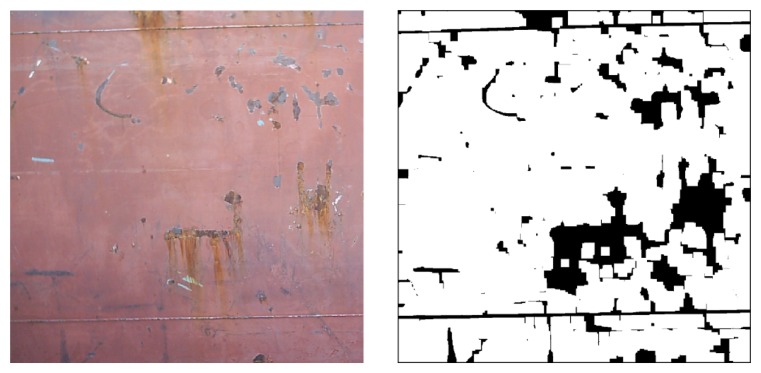
The result of UBE method after to process on a slice of 2 × 2 m of ship's hull.

Figure 13 shows the result of UBE method after to process on a slice of 2 × 2 meters of ship's hull. In this image we can observe that the corner down-left of the hull has poorer illumination than the upper part. The UBE method offered a performance superior to 90% in locating defects under different illumination conditions [17].

#### 3.2.4. Communication System

The communication system manages various standards that are used by the different levels within the system for information exchange. Table 1 shows the communication system's protocols and physical supports used by the different units. Table 2 shows the Communication modes used between every unit and its corresponding system and the standards used by the different systems and units during the ship's hull cleaning process.

### 3.3. Control Unit

The Control Unit is in charge of controlling the commands given to the engines to position the primary and secondary structures, and to activate the different mechanical elements involved in the grit blasting works.

When the Manual mode is activated, the cleaning system is controlled by the HMI system. This is not the usual way of working, however it is necessary to perform the motion, speed and position tests of the XYZ Table and to check that these movements are performed in a safe way, without a risk of accident. The Manual mode allows establishing the optimal values of the setup parameters that can be modified by the system when the Supervisor mode is activated in the Supervision Unit.

When the Automatic mode is activated, the Control Unit receives, through the communication system, the blasting dots matrix from the Inspection Unit, and the activation and deactivation of the grit flow. This unit turns the blasting dots matrix into the mechanical actions required to perform the grit blasting process.

The Control Unit is formed by the following elements

- **Communication system.** It interfaces the different elements of the Control Unit. The communication is established through a PROFIBUS DP bus.
- **HMI system.** This panel allows the operator to interact with the system by means of a graphic interface programmed with the PROTOOL tools. This system is composed by a panel operator, which allows to make basic manoeuvring along the hull surfaces (*i.e.*, to make movements in *X*, *Y* and *Z*, to calibrate positions and speeds in *X*, *Y* and *Z* to activate suction hood, *etc.)*.
- ***Control system.*** *The control system is where the control logic of the XYZ Table is executed to carry out the movements of the effectors that compose the cleaning. It is mainly composed by: a PLC, I/O DP modules, PROFIBUS DP communications modules, controllers and actuators.*
- ***Engineering station:*** *it carries out the reprogramming tasks of the elements within the bus, like ultrasonic sensor, DP (Decentralized PROFIBUS) I/O modules, PLC, SERVO-MOTORs, PROFIBUS communications*, etc.

For further information on these elements, see [18].

### 3.4. Teleoperated Unit

An added value to this system is the development of a wireless remote operation system that helps the operator to perform the visual inspection of the area to be cleaned. The remote operation station provides complete access to the software described in this section. The system consists of an industrial mobile panel and an industrial wireless access point; both elements are resistant to the aggressive conditions of the environment. The software developed for this system allows the operator to mark the cleaning spots or areas in the image of the ship surface using a mobile panel. When the Automatic mode is activated, the mobile panel displays the potential cleaning spots to the operator so he can verify them before the blasting process starts.

### 3.5. Cleaning and Recycling Unit

**Figure 14.**
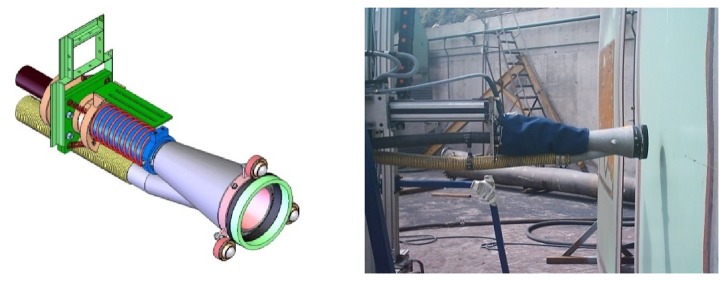
Blasting and suction hood.

The Cleaning and Recycling Unit consists of three main elements:

- **The Blasting and Suction system.** This element has been already described in this Section (Figure 14).
- **Grit blasting cut-off system.** The spotting process entails the interruption of the grit flow when the cleaning head has completed the cleaning works on a specific area or spot. To perform such interruption, a system has been designed to divert the grit flow to the recycling system during the change of position of the cleaning head. The grit jet cut-off device is controlled by the Control Unit, by activating two pneumatic electrically-operated valve.
- **Wastes recycling system.** This is a separate equipment that is integrated by the Control Unit through a simple protocol of signals interchange.

### 3.6. A Brief Evaluation of the Performance of the XYZ Table equipped with the Visual Inspection System

The design based on the XYZ Table performed inspection scans according to the size of the ship's surface with the maximum *X*×Y within the frame of the table (the largest prototype had a 1.6 × 2 m^2^ surface). The inspection procedure along the ship's hull consisted in a repetitive process: first, the XYZ Table was positioned on the ship's hull, the device took a picture of the area to be cleaned, the image was processed, the areas to be cleaned were selected and finally the grit blasting cleaning process started to be performed on the inspected surface.

The abovementioned solution based on using a XYZ Table as a secondary structure was finally implemented and verified in the Navantia shipyards facilities in Cartagena and Ferrol (Spain). The high positioning speed of this structure calls for the use of XYZ Table for cleaning little-damaged hull areas (see Table 3).

XYZ Table proved to be highly efficient for medium size ships with a 30% to 60% damaged hull surface, and for big size ships with a 10% to 20% damaged hull surface.

## 4. High Performance Sensorized Industrial System for Cleaning Ship's Hulls (HPSISC)

**Figure 15.**
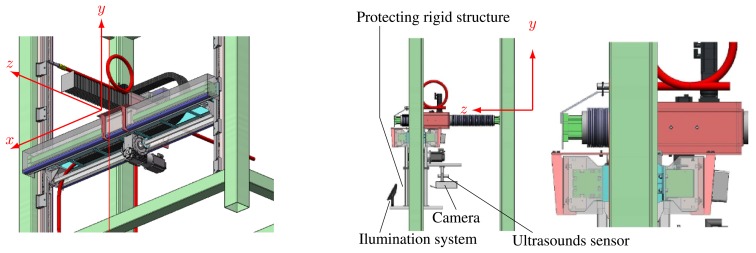
A CAD diagram of the new secondary structure.

In order to enhance the functionality of the system based on the XYZ Table by minimizing the blasting time used for cleaning the whole surface of a ship, a new secondary positioning structure design has been developed to achieve a better optimization of the blasting procedure. The XYZ Table has been replaced as a secondary structure by a mechanical positioning structure similar to the one shown in Figure 15. The purpose of the new design was (1) to reduce the time used by the XYZ Table for the positioning and vertical areas grit-blasting operations; and (2) to eliminate the expensive assembly of the secondary structure.

The new structure moves vertically along the *Y* -axis and on rails fitted on the external beam. This structure consists of the following elements:

- A linear axis of 2,750 mm that moves the blasting nozzle along the *X*-axis.
- A stainless steel and aluminum, 3,600 × 820 × 500 mm^3^ protection shield plated with easy to mount and dismount galvanized steel plates, designed to protect the linear axis and its engine from the abrasive material produced during the grit blasting procedure.
- A linear *Z*-axis of 660 mm that moves the blasting nozzle close or away from the ship's hull.
- A vision camera and an ultrasound sensor with protecting cap.

**Figure 16.**
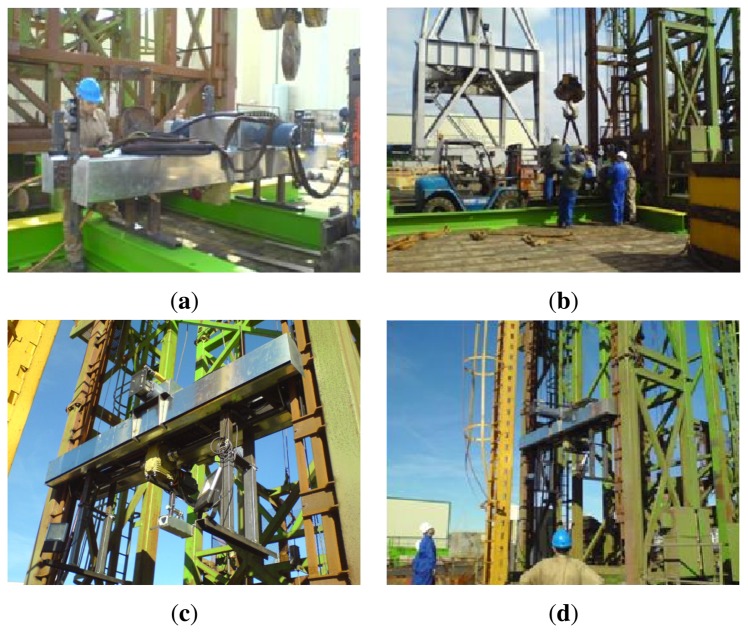
Assembly of the prototype on the shipyard's cranes. (**a**) New secondary structure; (**b**) and (**c**) Coupling operations carried out on the primary structure; (**d**) Final appearance of the primary and secondary structures after the coupling operations.

Figure 16 shows the different stages followed at the shipyard to mount the new structure.

The new design changes the strategy completely; now, the inspection scan can be carried out by means of surfaces' areas of *X*-length, the same as the linear axis of the new secondary structure, and *Y* length, the same as the primary structure. In this case, the process to be followed is the one shown in Table 4.

The main differences of this system with the system based on the XYZ Table are the following:

(1) the coupling/decoupling operations of the XYZ Table on/from the primary structure are eliminated;
(2) the image capturing process takes place on a whole area of the ship; and (3) the operator does not need to be present while the cleaning process of the ship's area takes place.

For the new HPSISC system, the process is the following: First, the secondary structure is placed on the bottom surface of the ship's hull, the protection shield is removed from the visual system and a picture of the area to be cleaned is taken. Then, the Control system saves the information obtained from such area and enters the necessary commands to make the secondary structure climb above the preceding position, right where the visual field of the camera ended. Next, a new picture of the ship's hull is taken and then, the secondary structure climbs upwards in the same way as before. The whole process is completed when the new structure reaches the top surface of the ship.

The processing system concatenates all the images obtained from the ship's hull and creates a single final image that covers the whole ship's column, from the bottom to the top, in the *X*-width. Therefore, a new vision algorithm had to be developed for this new process (Section 4.1); this algorithm is based on correlation operations and it creates the final image removing any potential overlap and correcting the mistakes caused by the calculations made with the movement equations of the secondary structure, or the mistakes produced by suspected shakes in the positioning mechanical system.

When the system receives the visual information obtained from the vertical area of a specific surface of the ship, then the inspection surface is analyzed and the areas where the cleaning process is going to be performed are manually or automatically selected. The UBE algorithm has been this time replaced by a better performance algorithm based on transformed wavelets.

After marking the areas to be cleaned on the final image, the processing system creates the blasting dots matrix required by the Control Unit to carry out the cleaning works.

While the operator, or the automatic detection system, selects the areas where grit blasting is going to be performed, the secondary structure returns to the original bottom position. Before starting the grit blasting works, the camera, the sensors and the engines of the secondary structure axes must be protected from any potential damage. Then, the blasting process starts, controlled in the same manner as described for the XYZ Table architecture.

The procedure implemented for the HPSISC system introduces important improvements. First, the automatic selection of the grit blasting areas made by the algorithm based on transformed wavelets is very similar to the manual selection made by any operator. These improvements provide the Automatic mode of the system with a greater reliability. Second, the grit blasting process is now carried out on a single area of the surface, whose width and height is the same as the displacement range of the new secondary structure and the primary structure, respectively. This way, the positioning time required by the former secondary structure (XYZ Table) is eliminated and the general cleaning process is accelerated.

**Figure 17.**
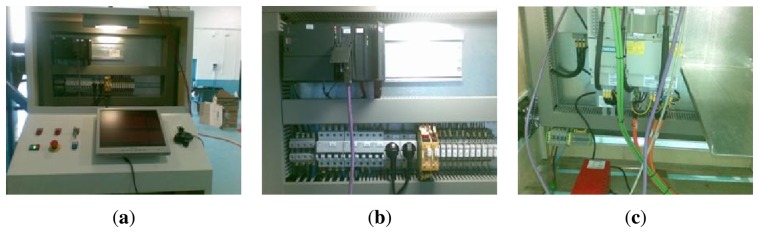
Communication Control and Electromechanical System Control consoles. (**a**) HMI System; (**b**) Control System; (**c**) servo-driver.

With regard to hardware, the visual displays have been improved as well as the electromechanical consoles that contain all the sensors and actuators controls of the system (see Figure 17). The safety of the operators has been improved too; now they can work sheltered by a protection cabin that is attached to one of the sides of the primary structure and is out of reach of any abrasive element.

### 4.1. Correlation Algorithm

As it was previously described, the new secondary structure is designed such that it can cover a whole vertical inspection area in only one inspection process. To achieve this, the secondary structure moves from the lower area to the upper area of the vertical inspection area in different steps with a distance of Δ*y*. On each step the inspection area that is covered is 2,750 mm (width) × 17,500 mm (height). Obviously, the width is associated to the length (*X* axis) of the secondary structure and the height is associated to the height covered by the image acquisition system from the position where the camera is located.

Therefore, when the system is covering the vertical surfaces and taking snapshots at different steps of the vertical structure, the Δ*y* value that should be associated to the vertical movement of the secondary structure should be 17,500 mm. However, this Δ*y* value has the risk that if the system does not have a perfect synchronized positioning system, or some oscillation or movement occurs when positioning, for example due to wind, some parts of the global image can be lost.

**Figure 18.**
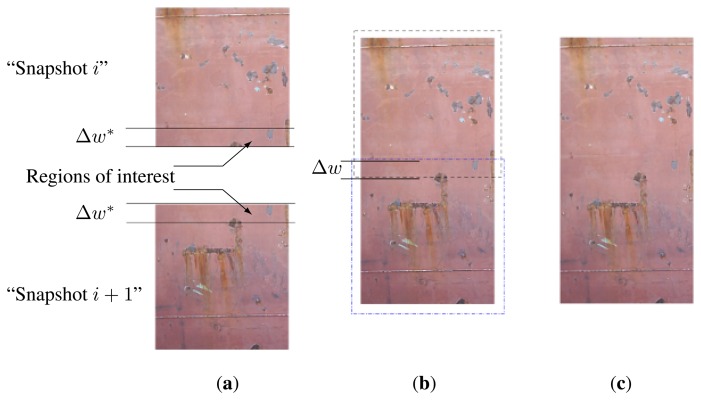
Consecutive image acquisition process when covering the vertical inspection area. (**a**) Input Image Snapshots; (**b**) Overlapping process; (**c**) Overlapping Output Image.

In order to avoid this loss of information, the Δ*y* value is reduced to 17,000 mm. In this case, as indicated in Figure 18, two consecutive images (“Snapshot *i*” and “Snapshot *i* + 1”) will share some parts, which need to be processed and removed when creating the global image that has to be shown to the operator via the graphics interface. This overlapped area is associated to a Δ*w* height value in each snapshot image acquired by the vision system. This Δ*w* area will be located in the upper part of the “Snapshot *i*” image, and in the lower part of the “Snapshot *i* + 1” image.

Therefore, an additional image processing algorithm was designed to remove this overlapped area when joining the two images. The elimination process is summarized in Figure 18. Figure 18a shows the two images (“Snapshot *i* ” and “Snapshot *i* + 1”) acquired by the vision system. In each image, a region of interest using a band of height Δw* is defined for each image. In the case of the “Snapshot *i* ” image, the band is defined in the upper area, and for the “Snapshot *i* + 1” image it is defined in the lower area. This Δw* is higher than the estimated Δ*w* value so that the image processing algorithm can compute the real Δ*w* value in a correlation process performed by evaluating correlation between these regions of interest. The highest correlation value is associated to the real Δ*w* value (Figure 18b). Then, a new global image overlapping this Δ*w* band is created (Figure 18c). This global image will be the new “Snapshot *i* ” image in the next step of the vertical movement. When the vertical movement ends, this processed global image will be offered to the operator, via the graphical interface of the system, so that he can select the areas that need to be cleaned along the whole vertical surface.

### 4.2. Entropy-Based Method for the Automatic Selection of the Wavelet Decomposition Level

The new design of the cleaning system, covering the whole ship's column, allowed to apply computational algorithms with higher computational requirements. The time required to perform a full capture of a ship's column permitted to apply texture analysis algorithms for defect detection. To this end, we developed a new algorithm for detecting defects based on the wavelet transform [19].

The suitability of wavelet transforms for use in image analysis is well established: a representation in terms of the frequency content of local regions over a range of scales provides an ideal framework for the analysis of image features, which in general are of different size and can often be characterized by their frequency domain properties [20]. For an image *f*(*x*, *y*) of size *M* × *N* pixels, each level of wavelet decomposition is obtained by applying two filters: a low-pass filter (L) and a high-pass filter (H). The different combinations of these filters produce four image that are here denoted with the subscripts LL, LH, HL and HH. In the first decomposition level (*j* = 1) four subimages or bands are produced: one smooth image, also called approximation, $f_{LL}^{\left(1\right)}(x,y)$, that represents an approximation of the original image *f*(*x*, *y*), and three detail subimages $f_{LH}^{\left(1\right)}(x,y)$, $f_{HL}^{\left(1\right)}(x,y)$ and $f_{HH}^{\left(1\right)}(x,y)$, which represent the horizontal, vertical and diagonal details respectively.

This makes the wavelet transform an attractive option when attempting defect detection in textured products, as reported by Truchetet [21] in his review of industrial applications of wavelet-based image processing. He reported different uses of wavelet analysis in successful machine vision applications: detecting defects for manufacturing applications for the production of furniture, textiles, integrated circuits, *etc.*

The review of the literature shows two categories of defect detection methods based on wavelet transform. The first category includes direct thresholding methods [22,23], whose design is based on the fact that texture background can be attenuated by the wavelet decomposition. If we remove the texture pattern from real texture, it is feasible to use existing defect detecting techniques for non-texture images, such as thresholding techniques [24]. Textural features extracted from wavelet-decomposed images are another category that is widely used for defect detection [25,26]. Features extracted from the texture patterns are used as feature vectors to feed a classifier (Bayer, Euclidean distance, Neural Networks or Support Vector Machines), which has unavoidable drawbacks when dealing with the vast image data obtained during inspection tasks. For instance, proximity-based methods tend to be computationally expensive and there is no straightforward way of defining a meaningful stopping criterion for data fusion (or division). Often, the learning-based classifiers need to be trained by the non-defect features, which is a troublesome and usually time consuming procedure, thus limiting its real-time applications [22]. For this reason we have focused on direct thresholding methods and we developed a new entropy-based algorithm for the automatic selection of the wavelet decomposition level and detection of defects on hull's surface.

**Figure 19.**
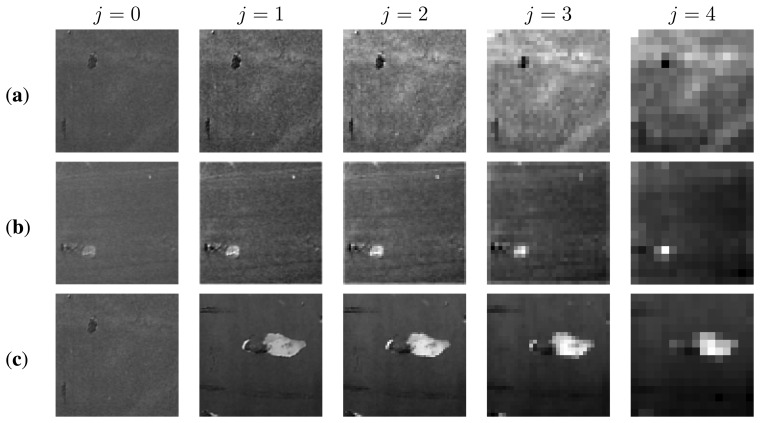
Approximation subimages $\left(f_{LL}^{\left(j\right)}(x,y)\right)$ of four wavelet decomposition levels for different images ((**a**) H225; (**b**) H34; and (**c**) H137) from portions of ship's hulls.

Figure 19 shows how the texture pattern degrades as the decomposition level increases. This degradation is distributed among the different decomposition levels depending on the texture nature and can be quantified by means of the Shannon entropy. The Shannon entropy function was used to identify the resolution level that provides the most information about defects in real textures. The Shannon entropy function [27,28] is calculated according to the expression:

$$s(X)=-\sum_{i=1}^{T}p(x_{i})\log p(x_{i})$$

where *X* = {*x*_1_, *x*_2_, …, *x_T_*} is a set of random variables with *T* outcomes and *p*(*x_i_*) is the probability of occurrence associated with *x_i_*.

For a 256 gray-level image of size *N_t_* pixels, we define a set of random variables *X* = {*x*_1_, *x*_2_, …, *x_i_*,…, *x*_256_} as the number of pixels in the image that have a gray level *i*. The probability of this random variable *x_i_* is calculated as the number of occurrences, *hist* [*x_i_*], divided by the total number of pixels *N_t_*:

$$p(x_{i})=\frac{\text{hist}\;[x_{i}]}{N_{t}}$$

To calculate the value of the Shannon entropy on the approximation subimage $\left(f_{LL}^{\left(j\right)}(x,y)\right)$ and on the horizontal, vertical and diagonal detail subimages $\left(f_{LH}^{\left(j\right)}(x,y\right)$, $f_{HL}^{\left(j\right)}(x,y)$, and $f_{HH}^{\left(j\right)}(x,y))$ in each decomposition level *j*, we obtain first the inverse wavelet transform of every subimage and then we apply these expressions:

$$\begin{array}{ll} S_{S}^{\left(j\right)} & =\frac{1}{N_{j}^{\text{pixels}}}\sum_{x}\sum_{y}s\;\left[W^{-1}\;\left[f_{LL}^{\left(j\right)}(x,y)\right]\right]\\ S_{h}^{\left(j\right)} & =\frac{1}{N_{j}^{\text{pixels}}}\sum_{x}\sum_{y}s\;\left[W^{-1}\;\left[f_{LH}^{\left(j\right)}(x,y)\right]\right]\\ S_{\upsilon }^{\left(j\right)} & =\frac{1}{N_{j}^{\text{pixels}}}\sum_{x}\sum_{y}s\;\left[W^{-1}\;\left[f_{HL}^{\left(j\right)}(x,y)\right]\right]\\ S_{d}^{\left(j\right)} & =\frac{1}{N_{j}^{\text{pixels}}}\sum_{x}\sum_{y}s\;\left[W^{-1}\;\left[f_{HH}^{\left(j\right)}(x,y)\right]\right] \end{array}$$

where $N_{\text{pixels}}^{j}$ is the number of pixels at each decomposition level *j*. We designate *W*^−1^ as the inverse transform wavelet of a subimage $f_{LL}^{\left(j\right)}(x,y)$ from the resolution level *j* to level (*j* −1) and we recursively invocate this function until *j* = 0.

To determine this optimal decomposition level, we use a ratio *R_j_*:

(1) $$R_{j}=\frac{S_{S}^{j}}{S_{S}^{j}+S_{h}^{j}+S_{\upsilon }^{j}+S_{d}^{j}},j=1,2,\ldots$$

*R_j_* indicates how much information about the texture pattern is contained in decomposition level *j*. Variations in this ratio allow detecting changes in the amount of information about the texture pattern between two consecutive decomposition levels. The goal is to find the optimal decomposition level that provides the maximum variation among two consecutive *R_j_* values, because this indicates that in decomposition level *j*, the texture pattern still present in level *j* − 1 has been removed, keeping useful information (defects). Once the optimal decomposition level is obtained, the process ends with the

production of the reconstructed image by the expression $F(x,y)=W^{-1}\;\left[f_{LL}^{\left(j\right)}(x,y)\right]$ and a binarization stage.

**Figure 20.**
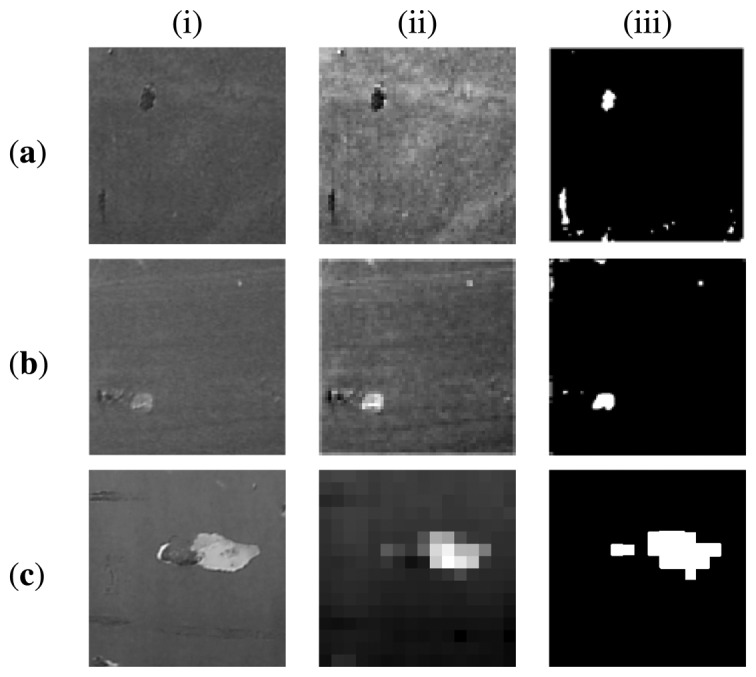
Column (**i**) shows texture images of portions of hulls; column (**ii**) shows reconstructed images resulting from the proposed reconstruction scheme; and column (**iii**) shows the resulting binarized images. Image Rows: (**a**) H225; (**b**) H34; (**c**) H137.

As can be observed in the Figure 20, the proposed method allowed detecting defects under different texture types on hull's surfaces. The proposed entropy-based algorithm achieved a higher performance than the UBE method (higher 95%).

### 4.3. Tests Carried out in the Shipyard's Facilities

**Figure 21.**
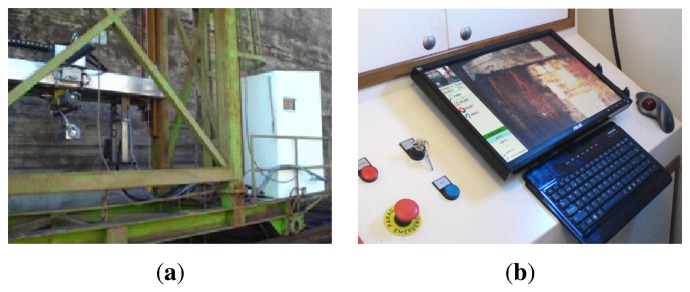
(**a**) Assembly of the system on the dry dock and view of the control cabin; (**b**) Detailed image of the Supervision Unit graphic interface installed inside the cabin.

After assembling the sensorized system, it was placed on the dry dock and the performance of the corresponding tests started. Figure 21 shows the system working on a wall of the shipyard. Figure 21a shows the system working during the grit blasting works; Figure 21b shows the enhanced graphic interface of the Supervision Unit. The software architecture of this new interface is similar to the architecture described in Section 3.4, but adapted for a new system that works on larger surfaces. The new interface permits the operator to carry out the usual selection of the areas to be cleaned.

**Figure 22.**
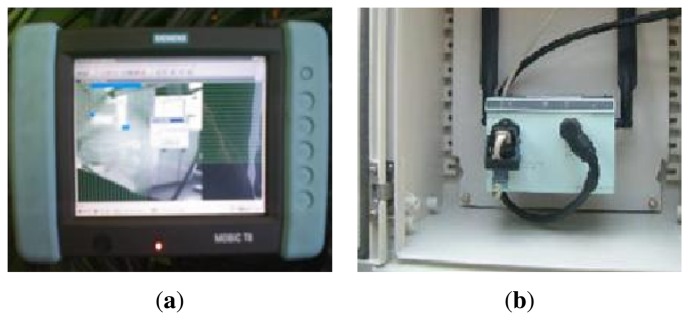
Remote wireless console to work outside of the cabin. (**a**) Panel that offers the same functions that the graphic interface installed inside the protection cabin. (**b**) The access point that is connected to the Control Unit and located at the cabinets of the primary structure to which the wireless console of the operator is remotely connected.

The possibility that the access to the cabin was not suitable enough or that the operator wanted to operate the system out of the cabin, from a place with a better view of the process, was taken into account. In order to satisfy these requirements, the system was equipped with a wireless remote control device, a Siemens Mobic panel, to be precise. Figure 22a shows the panel that offers the same functions that the graphic interface installed inside the protection cabin. Figure 22b shows the access point that is connected to the Control Unit and located at the cabinets of the primary structure to which the wireless console of the operator is remotely connected.

**Figure 23.**
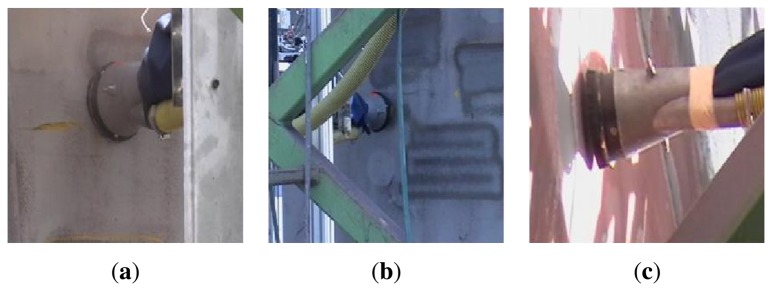
Grit blasting process performed on a vertical surface.

Finally, Figure 23 shows three pictures of the grit blasting process. Once the areas to be inspected from the remote wireless console or from the graphic interface installed inside the cabin have been verified, the Control Unit activates the engines that control the grit blasting nozzle. The right positioning of the grit blasting nozzle is favored by two factors: the effectiveness of the control and positioning algorithms developed and the effectiveness of the images creation and correcting algorithms implemented in the system. These algorithms have provided a good correlation between the data shown in the display of the operator remote console and the actual data obtained from the working surface of the grit blasting nozzle.

## 5. Conclusions

This article has given details on the progressive sensorization process that has been developed to automate one of the most important tasks performed in any ship-repair yards: the cleaning and preparation of the ship's hull before the repainting thereof. Different sensors have been developed to work on robotic devices built to satisfy the requirements of the repair-ship yards and with the usual elements kept in their work environment. These sensor systems are open systems that can be combined to meet the different automation needs, ranging from remote control to completely automatic operation, through supervised operations. The new sensor system developed (in particular the sensor described in Section 4) and introduced in this paper has complied with the specifications detailed at the beginning of Section 2. Besides, the development of these sensors has given rise to the following improvements:

- A new system that allows the automatic processing of large vertical surfaces of the ship's hull.
- Development of different algorithms to detect the areas to be repainted and implementation of procedures that allows taking the suitable decisions for treating the inspected surfaces. This procedure is especially useful to perform the spotting works because the work time has been minimized.
- Enhancement of the performance of manual operation, both in work time and in the amount of grit used during the cleaning. These improvements also entail a reduction in the costs and in the environmental impact.
- Enhancement of the operator's quality of work and reduction of the potential risk situations. The operator does not need to make much excessive physical effort while performing the grit blasting process anymore, so he can focus his efforts in supervising the process that is automatically carried out by the newly developed sensor system.
- The solution presented in this paper entails a new application of the artificial vision techniques in the ship repairing because they are integrated in the sensor system. Different image recognition algorithms have been tested on the various tasks usually performed in shipyards, and then validated and finally applied to a real environment.
- While designing the different cleaning techniques herein explained, new vision algorithms (defect detection, image chaining and image calibration) have been developed to detect defects on surfaces and to be applied to the adverse work conditions suffered in the shipyard: image capturing of operations carried out in open air, image distortion, camera movements, *etc.* Among these new algorithms, the most outstanding are: (1) thresholding based on Unsupervised Background Estimation; (2) Entropy-based method for the automatic selection of the Wavelet decomposition level; and (3) Image correction algorithm by means of an affine warping transform.

## Figures and Tables

**Table 1.** Level 1: Communication between units.

**UNITIES**	**Inspection**	**Control**	**Cleaning**	**Teleoperated**
**Supervision**	**Prot:** Shared Memory	**Prot:** Shared Memory	**Prot:** Shared Memory	**Prot:** TCP/IP
	**PhyS:** PROFIBUS DP	**PhyS:** PROFIBUS DP	**PhyS:** PROFIBUS DP	**PhyS:** Wifi a/b

**Inspection**		**Prot:** Shared Memory	NC	NC
		**PhyS:** PROFIBUS DP		

**Control**			**Prot:** Shared Memory	NC
			**PhyS:** PROFIBUS DP	

**Cleaning**				NC

**Prot**: Protocol. **PhyS**: Physical Support. NC: No Communication.

**Table 2.** Level 2: Communication among units and systems.

**Inspection Units**		
System	Protocol	Physical Support
Images capturing & processing	IEEE 1394	Fibre Optic
Images calibration	PCI	Frame Grabber
Defect detection	PCI	Frame Grabber

**Control Unit**		

System	Protocol	Physical Support
HMI	MPI	PROFIBUS DP
Control	Shared Memory	PROFIBUS DP
Engine Station	Property SIEMENS	PROFIBUS DP

**Cleaning Unit**		

System	Protocol	Physical Support
Blasting & Suction	Property SIEMENS	Wired
Grit-Blasting cut-off	NC	NC
Wastes Recycling	Shared Memory	PROFIBUS DP

**Table 3.** Performance of the cleaning procedure using the XYZ Table.

**Steps**		**Time**
(1)	The XYZ Table is coupled on the primary structure.	2.1 h
(2)	The primary structure is positioned on the dry dock.	0.58 h
(3)	Cleaning process.	
	(3.1.) The primary structure travels on the dry dock's rails.	
	(3.2.) The secondary structure travels to the cleaning area.	(F): 30 m^2^/h
	(3.3.) The Vision System captures and processes the area to be cleaned.	(S): 22 m^2^/h
	(3.4.) Blasting process.	
	Go back to (3.1.) until the cleaning process is completed.	
(4)	The structure is hoisted.	0.4 h
(5)	The XYZ Table is decoupled from the primary structure.	2.3 h

(F) Full-blasting; (S) Spot-blasting (over 15% damaged surface).

**Table 4.** Performance of the cleaning procedure using the HPSISC system.

**Steps**		**Time**
(1)	The primary structure is positioned on the dry dock.	0.6 h
(2)	Cleaning process.	
	(2.1.) The structure travels on the dry dock's rails.	
	(2.2.) The Vision System captures and processes the area to be cleaned.	(F): 71 m^2^/h
	(2.3.) Blasting process.	(S): 52 m^2^/h
	Go back to (2.1.) until the cleaning process is completed.	
(3)	The structure is hoisted.	0.4 h

(F) Full-blasting; (S) Spot-blasting (over 15% damaged surface).
